# Association of Health Literacy with Complementary and Alternative Medicine Use: A Cross-Sectional Study in Adult Primary Care Patients

**DOI:** 10.1186/1472-6882-11-138

**Published:** 2011-12-30

**Authors:** Sujeev S Bains, Leonard E Egede

**Affiliations:** 1 Department of Medicine, Medical University of South Carolina, Charleston, SC 29425, USA; 2 Center for Disease Prevention and Health Interventions for Diverse Populations, Ralph H Johnson VA Medical Center, Charleston, SC 29401, USA; 3 Center for Health Disparities Research, Medical University of South Carolina, Charleston, SC 29425, USA

## Abstract

**Background:**

In the United States, it is estimated that 40% of adults utilize complementary and alternative medicine (CAM) therapies. Recently, national surveys report that over 90 million adults have inadequate health literacy. To date, no study has assessed health literacy and its effect on CAM use. The primary objective of this study was to assess the relationship between health literacy and CAM use independent of educational attainment. Second objective was to evaluate the differential effect of health literacy on CAM use by race.

**Methods:**

351 patients were recruited from an outpatient primary care clinic. Validated surveys assessed CAM use (I-CAM-Q), health literacy (REALM-R), and demographic information. We compared demographics by health literacy (adequate vs. inadequate) and overall and individual CAM categories by health literacy using chi square statistics. We found a race by health literacy interaction and ran sequential logistic regression models stratified by race to test the association between health literacy and overall CAM use (Model 1), Model 1 + education (Model 2), and Model 2 + other demographic characteristics (Model 3). We reported the adjusted effect of health literacy on CAM use for both whites and African Americans separately.

**Results:**

75% of the participants had adequate literacy and 80% used CAM. CAM use differed by CAM category. Among whites, adequate health literacy was significantly associated with increased CAM use in both unadjusted (Model 1, OR 7.68; p = 0.001) and models adjusted for education (Model 2, OR 7.70; p = 0.002) and other sociodemographics (Model 3, OR 9.42; p = 0.01). Among African Americans, adequate health literacy was not associated with CAM use in any of the models.

**Conclusions:**

We found a race by literacy interaction suggesting that the relationship between health literacy and CAM use differed significantly by race. Adequate health literacy among whites is associated with increased CAM use, but not associated with CAM use in African Americans.

## Background

Complementary and Alternative Medicine (CAM) is defined by the United States National Center for Complementary and Alternative Medicine (NCCAM) as "a group of diverse medical and healthcare systems, practices, and products that are not presently considered to be part of conventional medicine" [1]. In the United States, it is estimated that 83 million adults [2,3] utilize CAM with total out-of pocket expenditures approaching $33.9 billion yearly [4].

CAM use is common among patients with medical illnesses [5-9] and has been associated with an increased number of chronic conditions [10,11], and greater healthcare utilization [12]. Studies also suggest that CAM use is related to socioeconomic variables such as higher educational attainment [6,13-16] and income status [15]. Higher CAM use has also been associated with health behavior such as lower medication adherence in certain populations [17-19]. In addition, CAM use has also been associated with health outcomes such as quality of life measures [20-23].

Research has also focused on health literacy and its relationship to chronic disease management and clinical outcomes [24-27]. Health literacy is defined as "the ability to access, understand, evaluate and communicate information as a way to promote, maintain and improve health in a variety of settings across the life-course." [28]. Recent national surveys report that over one-third of adults have limited health literacy [29]. Studies indicate that patient health literacy skills are associated with disease knowledge and self-management [24-27], medication adherence [30], quality of life [31,32] and clinical outcomes [27,33,34]. Studies also suggest that correlations exist between health literacy status and socioeconomic status [33,35-37]. In addition, evidence exists that educational attainment may not inevitably predict health literacy skills [25]. This is of importance since many population surveys that measure educational attainment do not include separate measures for health literacy.

In studies among patients with chronic illnesses, health literacy, along with CAM use, has been associated with health behavior. Examining the possible relationship between health literacy and CAM use may expand our understanding of behavioral determinants. Given research that CAM use may differ by race [3], analysis by racial categories may further delineate the overall impact of health literacy on CAM use. The knowledge of how these factors relate may benefit future interventional studies aimed at therapeutic targets that modify patient behavior. To our knowledge, few studies have assessed the relationship between health literacy status and CAM use. In addition, few studies have assessed the independent association between health literacy and CAM use by racial categories. The first objective of this study was to assess the association between health literacy and CAM use independent of educational attainment utilizing a valid CAM measure. The second objective was to evaluate the differential effect of health literacy on CAM use by race. Based upon prior literature, we hypothesized that adequate health literacy would predict increasing CAM use and further hypothesize that this relationship would differ by race and that whites with adequate literacy would have increased CAM use compared to African Americans.

## Methods

### Sample

351 patients at an adult primary care clinic of an academic medical center in the southeastern United States were recruited for the study. The clinic assesses adults aged eighteen and older with the patient volume remaining the same across most months. Study patients were recruited over a 10-week period in the summer months. The time frame for recruitment was chosen in line with a research course for undergraduate students undertaking research projects. Our sample size estimation was based on determining a difference between health literacy status (adequate vs. inadequate) and CAM use (type 1 error rate of 0.05). Consenting patients completed validated surveys to assess health literacy, CAM use, and socio-demographic information. Approval for the study was obtained from our Institutional Review Board.

### Demographic variables

Age was assessed as a categorical variable: 16-34 years, 35-49 years, 50-64 years, and 65 or greater years old. Race/ethnicity was categorized as white, black and Hispanic or other racial origin. Marital status was categorized as currently married and not married. Four levels of education were created: less than high school graduate, equal to high school graduate/no college, less than college graduate, and greater than college graduate. Employment status was categorized as currently employed and unemployed. Personal income was categorized as ≤ $10,000, ≤ $25,000, ≤ $50,000, < $75,000 and ≥ $75,000. Four categories of insurance status were created: private, Medicaid, Medicare, and uninsured. Health status was based on the individual's current opinion of his or her health in comparison to 12 months previously (better, same, or worse than 12 months ago). For this analysis, perceived health status was categorized as better, same and worse.

### Health literacy

We assessed health literacy by using the Revised Rapid Estimate of Adult Literacy in Medicine (REALM-R), an eight-item instrument designed to rapidly screen patients for potential health literacy problems. The REALM-R has been previously correlated with the Wide Range Achievement Test Revised (WRAT-R) (0.64) and a score of 6 on the REALM-R identified 26 of 30 persons scoring more than 1 standard deviation below the mean on the WRAT-R, corresponding to a sixth grade reading level" [38]. The REALM-R also has a shorter response burden than the WRAT-R and demonstrated a Cronbach's α of 0.91 [38]. The REALM-R is scored on a scale of 0 to 8 and asks patients to read a series of eight medical words, and a correct response is given for each correct pronunciation. These words include: osteporosis, allergic, jaundice, anemia, fatigue, directed, colitis and contipated. Scores of 6 or less correspond to a grade 6 reading level and identify patients at risk for poor health literacy [38]. For this analysis, health literacy was categorized as adequate health literacy (REALM-R > 6) and inadequate literacy (REALM-R ≤ 6).

### CAM use

We assessed CAM use using the International Questionnaire to Measure Use of Complementary and Alternative Medicine (I-CAM-Q) developed and tested by the National Research Center in Complementary and Alternative Medicine (NAFKAM) in Norway [39]. The I-CAM-Q asked patients if they had used herbs/herbal medicine, vitamins/minerals, and homeopathic remedies in the last 12 months. It also asked patients if they had used any of the following self-help practices in the last 12 months: meditation; yoga; qigong; tai chi; relaxation techniques; visualization, attending a traditional healing ceremony; or prayer for own health. For each type of CAM use, respondents were asked to indicate if they currently use it, main reason for last use, and to evaluate how helpful they found it [39]. For this analysis, CAM use was defined as using any of the above modalities or self-help practices.

### Statistical analysis

We performed three sets of analysis. First, we ran a confirmatory factor analysis to determine the factor structure of the REALM-R and calculated Cronbach's α for REALM-R to establish its internal consistency in our study population. Second, we compared sample demographics by health literacy status (adequate vs. inadequate), and compared overall CAM use and individual CAM categories by health literacy status using chi-squared statistics. Third, we tested and found a race by literacy interaction in our analysis. We subsequently ran sequential logistic regression models stratified by race to test the association between health literacy and overall CAM use (Model 1), health literacy and overall CAM use controlling for education (Model 2), and health literacy and overall CAM use controlling for both education and other demographic characteristics (Model 3). We only report significant findings for both whites and African Americans separately because of the interaction. For each regression model, overall CAM use (meditation, yoga, tai chi, relaxation, visualization, traditional healing, prayer, herbal medicine, vitamins, and homeopathy) was the dependent variable, and health literacy was the primary independent variable with education and other demographic variables (age, sex, marital status, household income, and health status) sequentially added in the model as covariates. All analyses were performed with STATA version 10 (Stata Corp. LP, College Station, TX), and a two-tailed α of 0.05 was used to assess for significance. Variables were included in the model based on clinical relevance.

## Results

### Factor analysis, internal consistency and reliability

The principal component analysis yielded an 8-item Realm-R with one factor having a measured eigenvalue of 5.02 that accounting for 63% of the variance. The eigenvalues provide the variance explained by each factor. Factor loadings ranged from 0.77 to 0.82. All individual items correlated at greater than 0.75 with this factor [40].

An item analysis for the 8-item Realm-R revealed a Cronbach's α of 0.91. The item-test correlation of the 8-item Realm-R ranged from 0.79 to 0.84 and the item analysis showed that alpha would not be meaningfully improved by dropping any one item from the scale.

### Overall sample characteristics

A total of 351 men and women were invited and recruited. 347 (98.8%) participants completed the surveys. 142 respondents (41%) were African American. Table 1 shows the demographics of the study by health literacy status. In the study, 75% of the patients had adequate health literacy and 25% had inadequate health literacy. Participants with adequate literacy were more likely to be over 50 years old(50+; 55.3%), white (68.8%), female (69%), married (55%), college graduate or higher (51.2%), employed (61.6%), have a household income > $75,000 (31.2%), have private insurance (67.1%), and reported their health status as better or same as last year (84.2%).

**Table 1:** Sample Characteristics by Health Literacy Levels

Characteristics (n = 347)	Adequate Health Literacy (REALM-R > 6)	Inadequate Health Literacy (REALM-R < 6)	P-value
	260(74.9)	87(25.0)	
Age Categories			
16-34 years	23.1	12.8	0.120
35-49 years	21.6	20.9	
50-64 years	32.6	44.2	
65 + years	22.8	22.1	
Race			
Black	27.7	82.7	< 0.001*
White	68.8	14.9	
Hispanic/Other	3.5	2.3	
Gender			
Male	31.0	36.0	0.387
Female	68.9	63.9	
Marital status			
Currently married	55.0	32.5	< 0.001*
Not married	44.9	67.5	
Education categories			
Less than high school graduate	8.3	23.8	< 0.001*
High school graduate	17.8	47.6	
Less than college graduate	22.6	20.2	
College graduate or higher	51.2	8.3	
Employment status			
Currently employed	61.6	43.5	0.003*
Unemployed	38.4	56.5	
Income categories			
< $10,000	10.9	30.5	< 0.001*
$10,000-< $25,000	13.4	42.7	
$25,000-< $50.000	24.3	18.3	
$50,000-< $75,000	20.2	6.1	
$75,000 +	31.1	2.4	
Insurance status			
Private	67.1	36.1	< 0.001*
Medicaid	7.5	18.1	
Medicare	20.2	27.7	
Uninsured	5.2	18.1	
Health status			
Better or same as last year	84.2	87.1	0.527
Worse than last year	15.8	12.9	

* p <*0.05*

### Sample characteristics by health literacy

In our sample, there were significant differences in health literacy level by demographic characteristics. Adequate health literacy was higher among white and Hispanic/other race compared to black race (93% white, 82% Hispanic/other versus 49% black; p < 0.001), and also higher among married (84% married versus 67% unmarried; p < 0.001), employed (81% employed versus 67% unemployed; p = 0.003), and the ones who had health insurance (85% privately insured versus 46% noninsured; p < 0.001). Adequate health literacy was higher among those with higher educational attainment (95% > college graduate versus 51% < high school graduate; p < 0.001), and higher household income (98% > $75,000 versus 52% < $10,000; p < 0.001), Gender and perceived health status were not statistically different by health literacy level.

### Health literacy and different types of CAM use

Health literacy status (adequate vs. inadequate) varied by category of CAM use. Table 2 displays health literacy status and the different types of CAM use. In our overall sample, 80% of the participants used CAM. Categorized by health literacy levels, 82% of those with adequate health literacy used CAM, while 74% of those with inadequate health literacy used CAM and this difference did not attain statistical significance. By CAM use category: 17% used meditation (14% adequate vs. 24% inadequate; p = 0.03) and 56% used vitamins (61% adequate vs. 42% inadequate; p = 0.003) which were both statistically significant.

**Table 2:** Health Literacy and different types of CAM use

Types of CAM use	All (%)	Adequate Health Literacy (%)	Inadequate Health Literacy (%)	P value
Overall CAM use	278(80.1)	214(82.3)	64(73.6)	0.077
Vitamins	192(56.3)	156(60.9)	36(42.4)	0.003*
Prayer	177(51.6)	126(49.2)	51(58.6)	0.130
Relaxation	60(17.8)	44(17.5)	16(18.6)	0.810
Meditation	57(16.7)	36(14.1)	21(24.1)	0.030*
Herbal Medications	46(13.7)	32(12.8)	14(16.5)	0.396
Yoga	22(6.4)	19(7.4)	3(3.5)	0.201
Visualization	19(5.6)	17(6.7)	2(2.3)	0.129
Homeopathy	14(4.3)	9(3.7)	5(6.1)	0.348
Tai Chi	3(0.9)	1(0.4)	2(2.4)	0.094
Attended traditional healing ceremony	3(0.9)	1(0.4)	2(2.4)	0.092

* p <*0.05*. Percentage includes all respondents.

Fifty-two percent used prayer (49% adequate vs. 59% inadequate), 18% used relaxation (18% adequate vs. 19% inadequate), 14% used herbal medications (13% adequate vs. 17% inadequate), 6% of the sample used yoga (7% adequate vs. 4% inadequate), 5% used visualization (7% adequate vs. 2% inadequate), 4% used homeopathy (4% adequate vs. 6% inadequate) < 1% used tai chi (0.4% adequate vs. 2% inadequate), < 1% attended traditional healing (0.4% adequate vs. 2% inadequate), and which were not statistically significant. No subjects used qigong as a CAM modality and subsequently this modality was excluded from our analysis.

Table 3 shows sequentially built logistic models of CAM use. Our analysis did show a significant race literacy interaction and subsequent testing of our separate models by racial category (Table 3) revealed that adequate health literacy among whites was associated with increased CAM use (Model 1, OR 7.68; 95% CI 2.35-25.1, p = 0.001). Adequate health literacy among whites remained significantly associated with increased CAM use after controlling for education (Model 2, OR 7.70; 95% CI 2.14-27.7, p = 0.002) and other demographics (Model 3, OR 9.42; 95% CI 1.66-53.5; p = 0.01). Adequate health literacy among African Americans was not significantly associated with CAM use (Model 1, OR 1.15; 95% CI 0.50-2.64; p = 0.74 and remained not significantly associated with CAM use after controlling for education (Model 2, OR 0.86; 95% CI 0.33-2.23; p = 0.76) and socio-demographics (Model 3, OR 0.97 95% CI 0.27-3.48; p = 0.96).

**Table 3:** Sequentially-Built Logistic Models of CAM use by racial category*

	White (n = 187)		Black (n = 133)	
	Odds Ratio (95% CI)	P-value	Odds Ratio (95% CI)	P-value
Model 1: Adequate Health Literacy Only (Ref: Inadequate Health Literacy)	7.68 (2.35,25.1)	0.001	1.15 (0.50,2.64)	0.735
Model 2: Adequate Health Literacy and Education Only (Ref: Inadequate Health Literacy)	7.70 (2.14,27.7)	0.002	0.86 (0.33,2.23)	0.756
Model 3: Adequate Health Literacy and Socio-Demographics (Ref: Inadequate Health Literacy)	9.42 (1.66,53.5)	0.011	0.97 (0.27,3.48)	0.958

* Model 1-unadjusted. Model 2- adjusted for education category (< high school graduate, high school graduate, < college graduate, college graduate+). Model 3- adjusted for socio-demographics (education category, age category, gender, income, employment status, marital status, and health status).

## Discussion

In our study we found a race by literacy interaction suggesting that the relationship between CAM use and health literacy differed significantly by race. Consistent with our hypothesis, our analysis by race revealed that CAM use was related to adequate health literacy among whites however, CAM use was not related to health literacy among African Americans. Among whites, the relationship between CAM use and adequate literacy remained significant after adjusting for educational attainment and other sociodemographic variables. Also, health literacy levels were higher among whites as compared to African Americans suggesting that adequate health literacy may predict overall CAM use. This is consistent with research that health literacy skills are associated with health seeking behaviors [41,42].

Our results also indicate that health literacy status differed by CAM use category. Specifically, adequate health literacy was associated with vitamin use while inadequate health literacy was associated with meditation use. One possible explanation for this discrepancy is that only a small portion of our patients used meditation compared to vitamins. The small sample of patients that used meditation (n = 57) may not reflect actual relationships in the population as a whole. Also, unmeasured patient level factors may have influenced our results. For instance, it is possible that patients with cormorbid illnesses and subsequent disease familiarity may be more apt to take pill based therapies compared to other CAM modalities based upon prior familiarity with medications. Larger population studies including patient level factors such as health knowledge and health literacy need to be conducted to accurately predict the independent relationship between health literacy and different CAM modalities.

Although national estimates indicate that whites are more likely use CAM than African Americans [2], previous national surveys lacked measures of health literacy. Factors such as health literacy may explain the propensity to use CAM in certain populations. In addition, although higher educational attainment has been associated with increased CAM use [6,13-16], years of schooling may not necessarily predict reading ability [25]. This suggests that both educational attainment and health literacy need to be included in studies assessing possible determinants of CAM use. In our study, among whites, reading ability related to utilizing CAM even after adjusting for educational attainment and other sociodemographic factors. One possible explanation is that CAM may attract white patients with increased reading skills regardless of years of schooling or socioeconomic status. An implication of this finding is that patients with improved reading skills may utilize CAM for health promotion or health education purposes. Studies including health literacy, CAM use and preventative care or disease self-management outcomes may provide insight into whether health literacy skills influence the relationship between CAM use and patient health behavior in this racial group. Of note, among African Americans, we did not find an association between health literacy and CAM use. This suggests that other factors may influence CAM use among African Americans. Given our findings, additional studies are needed to corroborate our findings and investigate the mechanism by which health literacy relates to CAM use by race.

This study has some limitations. The current study was cross-sectional in nature and cannot speak to temporal relationships or causality. Second, this study was conducted at a single academic center and it is possible that the subjects in this study were better educated and more likely to have adequate health literacy skills than those at community clinics or rural areas. It is also possible that patients in an academic center were more likely to use CAM secondary to having a higher number of chronic conditions. Third, although adapted and tested, the I-CAM-Q may require further validation and may not have included all CAM therapies utilized in our population. Furthermore, our analysis did not include reasons for CAM use. Additional analysis including reasons for CAM use may have assisted in understanding the distribution of CAM use in our population. Fourth, although the REALM-R is a valid measure of health literacy, it uses medical terms which may not reflect the ability of participants to read CAM related words. Fifth, the lack of effect of health literacy among African American respondents might have been the result of inadequate sample size. Sixth, our study was cross sectional and selection bias is always a possibility. The high response rate in our sample may have minimized the effect of this bias.

## Conclusion

Although previous studies have demonstrated that CAM use is associated with educational attainment [6,13-16], to our knowledge this is the first study to examine the association between health literacy and CAM use. In summary, we found a race by literacy interaction suggesting that the relationship between health literacy and CAM use differed significantly by race. We found that adequate health literacy among whites is associated with increased CAM use as compared to African Americans. This implies that racial differences exist regarding the relationship of health literacy with CAM use. Further research is needed to examine the possible mechanisms by which health literacy and CAM use are related. These studies should include analysis of reasons for CAM use in order to provide insight into what environmental factors contribute to CAM use. These efforts may help identify which internal and external factors in a diverse population increase the likelihood of CAM use.

## List of abbreviations

I-CAM-Q: International Complementary Alternative Medicine Questionnaire; REALM-R: Rapid Estimate of Adult Literacy in Medicine.

## Competing interests

The authors declare that they have no competing interests.

## Authors' contributions

SB contributed to the interpretation of the data, and directly involved in drafting the manuscript. LE was involved in the conceptual design and acquisition of the data, carrying out statistical analysis, and critical revision of content. All authors read and approved the final version of the manuscript.

## Pre-publication history

The pre-publication history for this paper can be accessed here:

http://www.biomedcentral.com/1472-6882/11/138/prepub

## References

[B1] National Center for Complementary and Alternative Medicine http://nccam.nih.gov/health/whatiscam/#1 Accessed May 22, 2010

[B2] Barnes PM Bloom B Nahin RL Complementary and alternative medicine use among adults and children: United States 2007 Natl Health Stat Report 2008 12 1 23 19361005

[B3] Tindle HA Davis RB Phillips RS Eisenberg DM Trends in use of complementary and alternative medicine by US adults:1997-2002 Altern Ther Health Med 2005 11 1 42 9 15712765

[B4] Nahin RL Barnes PM Stussman BJ Bloom B Costs of complementary and alternative (CAM) and frequency of visits to CAM practitioners: United States 2007 National health Stat Report 2009 18 1 14 19771719

[B5] Fairfield KM Eisenberg DM Davis RB Patterns of use, expenditures, and perceived efficacy of complementary and alternative therapies in HIV-infected patients Arch Intern Med 1998 158 20 2257 64 10.1001/archinte.158.20.2257 9818806

[B6] Egede LE Ye X Zheng D Silverstein MD The prevalence and pattern of complementary and alternative medicine use in individuals with diabetes Diabetes Care 2002 25 2 324 9 10.2337/diacare.25.2.324 11815504

[B7] Nayak S Matheis RJ Schoenberger NE Shiflett SC Use of unconventional therapies by individuals with multiple sclerosis Clin Rehabil 2003 17 2 181 91 10.1191/0269215503cr604oa 12625659

[B8] Montazeri A Sajadian A Ebrahimi M Akbari ME Depression and the use of complementary medicine among breast cancer patients Support Care Cancer 2005 13 5 339 42 10.1007/s00520-004-0709-z 15549425

[B9] Chong CA Diaz-Granados N Hawker GA Complementary and alternative medicine use by osteoporosis clinic patients Osteoporos Int 2007 18 11 1547 56 10.1007/s00198-007-0417-x 17603742

[B10] Millar WJ Use of alternative health care practitioners by Canadians Can J Public Health 1997 88 3 154 8 9260354 10.1007/BF03403879 PMC6990232

[B11] Ai AL Bolling SF The use of complementary and alternative therapies among middle-aged and older cardiac patients Am J Med Qual 2002 17 1 21 7 10.1177/106286060201700105 11852673

[B12] Li JZ Quinn JV McCulloch CE Patterns of complementary and alternative medicine use in ED patients and its association with health care utilization Am J Emerg Med 2004 22 3 187 91 10.1016/j.ajem.2004.02.006 15138954

[B13] Field KM Jenkins MA Friedlander ML Predictors of the use of complementary and alternative medicine (CAM) by women at high risk for breast cancer Eur J Cancer 2009 45 4 551 60 10.1016/j.ejca.2008.09.023 18996690

[B14] Barner JC Bohman TM Brown CM Use of complementary and alternative medicine for treatment among African-Americans: a multivariate analysis Res Social Adm Pharm 2010 6 3 196 208 10.1016/j.sapharm.2009.08.001 20813333 PMC2933406

[B15] Brown C Barner J Bohman T Richards K A multivariate test of an expanded Andersen Health Care utilization model for complementary and alternative medicine (CAM) use in African Americans J Altern Complement Med 2009 15 8 911 9 10.1089/acm.2008.0561 19678783 PMC3191375

[B16] Wyatt G Sikorskii A Wills CE Su H Complementary and alternative medicine use, spending, and quality of life in early stage breast cancer Nurs Res 2009 59 1 58 66 10.1097/NNR.0b013e3181c3bd26 20010046

[B17] Jernewall N Zea MC Reisen CA Poppen PJ Complementary and alternative medicine and adherence to care among HIV-positive Latino gay and bisexual men AIDS Care 2005 17 5 601 9 10.1080/09540120512331314295 16036246

[B18] Gohar F Greenfield SM Beevers DG Self-care and adherence to medication: a survey in the hypertension outpatient clinic BMC Complement Altern Med 2008 8 4 10.1186/1472-6882-8-4 18261219 PMC2259297

[B19] Krousel-Wood MA Muntner P Joyce CJ Adverse effects of complementary and alternative medicine on antihypertensive medication adherence: findings from the cohort study of medication adherence among older adults J Am Geriatr Soc 2010 58 1 54 61 10.1111/j.1532-5415.2009.02639.x 20122040 PMC2920063

[B20] Buettner C Kroenke CH Phillips RS Correlates of use of different types of complementary and alternative medicine by breast cancer survivors in the nurses' health study Breast Cancer Res Treat 2006 100 2 219 27 10.1007/s10549-006-9239-3 16821087

[B21] Hlubocky FJ Ratain MJ Wen M Daugherty CK Complementary and alternative 0 medicine among advanced cancer patients enrolled on phase I trials: a study of prognosis, quality of life, and preferences for decision making J Clin Oncol 2007 25 5 548 54 10.1200/JCO.2005.03.9800 17290064

[B22] Lawsin C DuHamel K Itzkowitz SH Demographic, medical, and psychosocial correlates to CAM use among survivors of colorectal cancer Support Care Cancer 2007 15 5 557 64 10.1007/s00520-006-0198-3 17205277

[B23] Yamada K Low level of quality of life in patients with mental and behavioral disorders wanting complementary and alternative (kampo) therapy Qual Life Res 2007 16 5 787 92 10.1007/s11136-007-9179-3 17295101

[B24] Williams MV Baker DW Parker RM Nurss JR Relationship of functional health literacy to patients' knowledge of their chronic disease. A study of patients with hypertension and diabetes Arch Intern Med 1998 158 2 166 72 10.1001/archinte.158.2.166 9448555

[B25] Williams MV Baker DW Honig EG Inadequate literacy is a barrier to asthma knowledge and self-care Chest 1998 114 4 1008 15 10.1378/chest.114.4.1008 9792569

[B26] Kalichman SC Rompa D Functional health literacy is associated with health status an health-related knowledge in people living with HIV-AIDS J Acquir Immune Defic Syndr 2000 25 4 337 44 10.1097/00126334-200012010-00007 11114834

[B27] Cavanaugh K Huizinga MM Wallston KA Association of numeracy and diabetes control Ann Intern Med 2008 148 10 737 46 18490687 10.7326/0003-4819-148-10-200805200-00006

[B28] Rootman I Gordon-el-Bihbety D A Vision for a Health Literate Canada. Report of the Expert Panel on Health Literacy 2008 Ottawa, ON: Canada Public Health Association

[B29] Kutner M Greenberg E Jin Y Paulsen C The health literacy of America's results: results from the 2003 national assessement of adult literacy 2006 Washington, DC: NCES. U.S. Dept. of Education

[B30] Gazmararian JA Kripalani S Miller MJ Factors associated with medication refill adherence in cardiovascular-related diseases: a focus on health literacy J Gen Intern Med 2006 21 12 1215 21 10.1111/j.1525-1497.2006.00591.x 17105519 PMC1924753

[B31] Mancuso CA Rincon M Impact of health literacy on longitudinal asthma outcomes J Gen Intern Med 2006 21 8 813 7 10.1111/j.1525-1497.2006.00528.x 16881939 PMC1831574

[B32] Bautista RE Glen ET Shetty NK Wludyka P The association between health literacy and outcomes of care among epilepsy patients Seizure 2009 18 6 400 4 10.1016/j.seizure.2009.02.004 19324575

[B33] Schillinger D Grumbach K Piette J Association of health literacy with diabetes outcomes JAMA 2002 288 4 475 82 10.1001/jama.288.4.475 12132978

[B34] Baker DW Wolf MS Feinglass J Health literacy and mortality among elderly persons Arch Intern Med 2007 167 14 1503 9 10.1001/archinte.167.14.1503 17646604

[B35] Kim S Love F Quistberg DA Shea JA Association of health literacy with self-management behavior in patients with diabetes Diabetes Care 2004 12 2980 2 10.2337/diacare.27.12.2980 15562219

[B36] Rothman R Malone R Bryant B The relationship between literacy and glycemic control in a diabetes disease-management program Diabetes Educ 2004 30 2 263 73 10.1177/014572170403000219 15095516

[B37] Shea JA Beers BB McDonald VJ Assessing health literacy in African American and Caucasian adults: disparities in rapid estimate of adult literacy in medicine (REALM) scores Fam Med 2004 8 575 81 15343419

[B38] Bass PF Wilson JF Griffith CH A shortened instrument for literacy screening J Gen Intern Med 2003 18 12 1036 8 10.1111/j.1525-1497.2003.10651.x 14687263 PMC1494969

[B39] Quandt SA Verhoef MJ Arcury TA Development of an international questionnaire to measure use of complementary and alternative medicine (I-CAM-Q) J Altern Complement Med 2009 15 4 331 9 10.1089/acm.2008.0521 19388855 PMC3189003

[B40] Nunnally JC Bernstein I Psychometric Theory 1994 New York: McGraw-Hill

[B41] Scott TL Gazmararian JA Williams MV Baker DW Health literacy and preventive health care use among Medicare enrollees in a managed care organization Med Care 2002 40 5 395 404 10.1097/00005650-200205000-00005 11961474

[B42] von Wagner C Knight K Steptoe A Wardle J Functional health literacy and health-promoting behaviour in a national sample of British adults J Epidemiol Community Health 2007 61 12 1086 90 10.1136/jech.2006.053967 18000132 PMC2465677

